# Exploring care-experienced young people’s perceptions of the use of administrative data in research

**DOI:** 10.23889/ijpds.v9i5.2530

**Published:** 2024-09-18

**Authors:** Jie Song, Charini Nanayakkara, Peter Christen, Eilidh Garrett

**Affiliations:** 1 The Australian National University; 2 The University of Edinburgh

## Objectives

Linkage of data containing personal information allows extensive studies in the health and social sciences for population level studies. However, real-world linkage applications often lack ground truth (GT) data, generally due to privacy concerns, which hinders linkage quality assessment. We propose a method to assess the linkage of sensitive population data in the absence of GT data.

## Approach

Our method takes as input a set of sensitive and public population clusters. The sensitive clusters are generated with some record linkage algorithm, whereas the public population dataset, which is considered an approximation for the GT, is assumed to have a group identifier allowing generation of known clusters, such as households or families. We encode clusters as bit vectors and conduct cluster matching across the sensitive and public datasets using vector comparisons. Based on matched clusters, the population linkage quality of sensitive data is estimated.

## Results

We conducted experiments on a sensitive Scottish birth data set containing 17,613 records from 1861 to 1901, and four successive publicly available Scottish census datasets from 1871 to 1901. Using the quality measures precision, recall and the F-measure, we show that our estimations are close to GT quality, with a high correlation in F-measure values ranging from 88% to 99%.

## Conclusion

We proposed a method to estimate the linkage quality of sensitive population data for situations where public data with similar characteristics are available for approximating the GT. Our experimental evaluation showed that our method was effective in estimating true linkage quality with high accuracy.

